# “Psychological Antibodies” to Safeguard Frontline Healthcare Warriors Mental Health Against COVID-19 Pandemic-Related Psychopathology

**DOI:** 10.3389/fpsyt.2020.590160

**Published:** 2020-12-18

**Authors:** Aishwarya Jaiswal, Tushar Singh, Yogesh Kumar Arya

**Affiliations:** Department of Psychology, Banaras Hindu University, Varanasi, India

**Keywords:** psychological antibodies, COVID-19 pandemic, psychopathology, psychological immunity, frontline healthcare warriors

The rapid and unprecedented worldwide spread of the novel coronavirus, also termed as 2019 novel coronavirus (2019-nCoV) or 2019 coronavirus disease (COVID-19), has immensely strained the existing healthcare systems (HCSs) throughout the world (1). The frontline healthcare workers (HCWs) (doctors, nurses, paramedics, ambulance personnel) are occupied with the direct diagnosis, treatment, and care of the COVID-19 infected patients and hold the significant responsibility of flattening the pandemic growth curve and reducing the infection fatality rate. Though HCWs would have their Behavioral Immune System continuously active during this pandemic situation (2), excessive workload, the risk of nosocomial transmission, lack of essential resources and specific medical treatment, and frequent encounters with trauma and death have heightened their risk of psychological distress (3) and trauma (4); psychopathology, such as substance use (2); mood disorders, such as insomnia, anxiety, and depression (3); delusional episodes; suicidality (4); and even suicide (5, 6). An eventual rise in the need of mental health services by HCWs is probable as these mental health consequences may remain even after the pandemic remits (7, 8). As the medical professionals are the most significant assets in countering the pandemic, safeguarding the physically and emotionally exhausted (9) HCWs' mental health becomes significant. This opinion article briefly describes the psychopathology encountered by HCWs during the 2019-nCoV pandemic and the protective role “psychological antibodies” constituting the psychological immunity (PI) can have in guarding HCWs against these psychopathological symptoms. Particular attention is drawn toward the need for developing evidence-informed individual- and organizational-level PI-boosting interventions for HCWs.

## COVID-19-Linked Psychopathology in HCWs

The medical personnel attending the COVID-19 patients report significantly higher symptoms of somatization, obsession, compulsion, anxiety, phobic anxiety, and psychoticism. Besides, they have significantly lower interpersonal sensitivity and overall poor mental health (10). HCWs also suffer emotional disturbances, such as anxiety and depression, excessive workload, physical and mental exhaustion, burnout, post-traumatic stress symptoms, loneliness, sleep disorders, and distress (3, 11–17). The fear of getting infected and having a sudden role reversal from a healthcare provider to a medical patient leads to the feelings of frustration and helplessness, adjustment issues, perceived stigma, and fear of discrimination (18). HCWs are performing duties outside of perceived skills, experiencing life threats, and witnessing co-worker's serious illness, injury, and death (19), all of which are specific factors that put them at a higher risk for developing post-traumatic stress disorder (PTSD) a few months later (20). A timely systematic review and meta-analyses provided evidence that a considerable fraction of HCWs experienced significant levels of anxiety, depression, and insomnia during the coronavirus pandemic (21). The frontline HCWs in the department of respiratory medicine, emergency, intensive care unit (ICU), and infectious disease have 2-fold chances of experiencing anxiety, depression, and other mental health problems compared with the non-clinical staff (15, 22, 23). The infection rate among medical staff (24), fear of infection to colleagues and family, protective measures, and medical violence further add to HCWs' psychological issues (25). These mental issues can potentially lead to medical treatment errors, patient mortality, substance abuse, and even suicidal ideation among HCWs. Thus, “Healing the Healer” (26) becomes crucial. In addition to these potential hazards that exceed the consequences of COVID-19 itself (27), the accumulated psychological pressure and the intense fear of death during the pandemic even pushed the already vulnerable medical professionals into committing suicide (5, 6). Though researchers (9, 17, 22) indicate a need for effective strategies, mental health informed interventions, and regular intensive training for HCWs, evidence-based evaluations and potential mental health interventions targeting frontline HCWs are relatively scarce (3, 28). Further, till date, neither any clinician-administered scale for measuring psychological distress or disorders in the COVID-19 context (29) nor any specific recommendations from the international bodies on the addressal of the mental health concerns during the pandemic are available (30). Identifying the personal resilience resources that mitigate stress can aid in the rapid design of evidence-informed individual- and organizational-level interventions for HCWs.

## Psychological Antibodies and the Psychological Immune System

The concept of the Psychological Immune System (PIS) was proposed by Olah (31) to integrate the isolated but empirically correlated character strengths and stress-resistant resources of the personality into one comprehensive system. PIS is a multidimensional yet integrated unit of personal resilience resources and adaptive capacities, also referred to as “psychological antibodies” (32), that provide immunity against damage, stress (33), and traumatic events (34). Table 1 briefly describes the three subsystems entailed in the PIS and their respective psychological antibodies. During the coping process, these subsystems dynamically interact and regulate one another's functioning and guide the person to use flexible and self-developing coping strategies (35, 36).

**Table 1:** Subsystems of the psychological immune system, their respective antibodies, and practical recommendations for enhancing healthcare workers' psychological immunity.

**PIS**	**Psychological antibodies**	**Description**	**Practical recommendations**
Approach-Belief Subsystem (ABS) (it guides an individual's orientation toward the environment)	Positive thinking	It involves cognitive personality dimensions facilitating the anticipation of positive outcomes in circumstances outside one's control.	- Stay optimistic and hopeful. - Practice some form of positive ideation pertaining to the current and upcoming circumstances.
	Sense of control	It is a sense of personal influence or perceived control over life events.	- Believe in your ability to change or modify your everyday occurrences and outcomes. - Do not attribute your failures to luck or chance factors.
	Sense of coherence	It is the belief that life is understandable, comprehensible, and manageable.	- Believe that the environment is predictable and the occurrences therein are manageable.
	Sense of self-growth	It is a stable conviction that one can continuously overachieve and enhance their personality and personal productions.	- View every circumstance as a growth-inducing experience that further nourishes your personality and productivity.
Monitoring-Creating-Executing Subsystem (MCES) (it initiates the search for information and puts into action the resources necessary to influence and create possibilities within the environment)	Creative self-concept	It is an individual's strong belief in their creative power, self-worth, and the worth of their life accomplishments.	- View difficult and unprecedented circumstances from a novel perspective and come up with creative solutions. - Maintain a high sense of self-worth. - Try to become a resourceful person.
	Self-efficacy	It is the ability to expect that one can act in the way required to produce the desired outcomes.	- Continually upgrade your knowledge and medical skills. - Hold confidence in your abilities.
	Goal orientation	It is the ability to maintain motivation and endurance in accomplishing tasks even in the face of adversities and obstacles.	- Develop intrinsic motivation towards tasks. - Remain endurant while completing any task howsoever challenging or tricky it appears.
	Problem-solving capacity	It is the ability to reconstruct and reorganize learned experiences to create new ideas and plans and execute alternative solutions for handling problems and difficulties.	- Enhance your innovative ability. - Indulge in constructive thinking.
	Change and challenge orientation	It involves openness to novel experiences, intrinsic motivation to explore the environment, sensitive perception following changes, and anticipation of change as adaptive, challenging, and positive.	- Do not refrain from novel circumstances. - Anticipate changes as adaptive and/or positive rather than as threats.
	Social monitoring capacity	It is the sensitive and selective observation and use of social or environmental information for achieving future aims.	- Develop mindful awareness. - Form interpersonal connections. - Develop empathetic ability. - Enhance your emotional intelligence.
	Social mobilizing capacity	It is the ability to motivate, force, govern, and direct human resources to benefit one's aims.	- Develop leadership abilities, communication skills, and social assertiveness.
	Social creating capacity	It is the ability to create social resources in situations where their existence is inevident.	- Stay vigilant about others' skills and talents. - Develop teamwork spirit.
Self-Regulating Subsystem (SRS) (it stabilizes emotions and ensures the functioning of the preceding subsystems)	Synchronicity	It is the ability to be “in flow” with the current environment or task and maintain a maximal concentration on personal and environmental issues.	- Practice mindfulness and try to maintain mindful attention. - Learn the skills (e.g., avoidance) needed to disengage from the distracting conditions.
	Impulse control	It is the ability to manage behavior by rational control over spontaneous actions.	- Control your impulsive reactions and behavior. - Orient yourself toward rational and reflective actions. - Thoroughly contemplate on any decision before acting on it.
	Emotion control	It is the ability to regulate the negative feelings and emotions (e.g., worry, anxiety, depression, etc.) induced by anticipation of failure.	- Do not carry the baggage of past setbacks or failures. - Instead of denying, accept your negative feelings and emotions.
	Irritability Control	It is the ability to control and constructively regulate impatience and anger stemming from unsatisfied essential needs.	- Do not lose your temper easily. - Develop frustration tolerance.

## Psychological Immunity: A Counter to COVID-19-Related Psychopathology

Prior studies assessing psychological immunity (PI) protective potentials (psychological antibodies) in high-stress occupation personnel, such as emergency nurses (37), medical professionals (32), and military soldiers (38), have yielded promising results. PI holds a strong positive correlation with life satisfaction and well-being dimensions (environmental mastery, purpose in life, personal growth, self-acceptance, positive relations, and autonomy) and a negative correlation with burnout (33). The antibodies, sense of control (SOC), sense of self-growth, synchronicity, impulse, emotion, and irritability control are strongly correlated with mental and physical health (33). Positive thinking, SOC, and sense of self-growth mediate the psychological adjustment–mental health linkage in instances of acute psychopathology (39). The personality resources comprising PIS significantly predict the level of satisfaction in gymnasts (40). Approach-Belief Subsystem (ABS) and Monitoring–Creating–Executing Subsystem (MCES) correlate positively with the hope of attaining goals, and the entire PIS correlates positively with life satisfaction and negatively with depression (41). There also exists a strong correlation between PI and life expectancy (42).

PI, and the psychological antibodies therein, can provide HCWs effective coping against stress and guard them against psychopathology. Positive thinking educational intervention and training via social media reduce nurses' job stress (43) and enhance their quality of work-life (44). It is plausible as positive thinking entails optimism and hopefulness, which influence the primary appraisal process and the perception of person-situation transactions. While low job control associates with increased sickness absence in hospital physicians (45), control over work and significant levels of autonomy bear a protective effect on mental health (46, 47). A study on female nurses found that SOC is a protective factor for depressive state, burnout, and job dissatisfaction and can be a health-promoting resource (48). A sense of self-growth promotes openness and assimilation of the new experiences and strongly motivates self-actualization and self-expansion. Personal growth has significant association with role boundary, role insufficiency, role ambiguity, and interpersonal, psychological, and physical strain (49). Thus, the HCSs shall maintain an increased workforce and ensure that their HCWs have well-defined duties, shorter working periods, flexible schedules, shift duties, regular breaks, and supervisor support. Studies with HCWs have shown that self-concept underlies the development of the professional self-concept (50). Hence possessing a high creative self-concept will enable HCWs to bear a positive outlook and success orientation toward most situations they encounter. Contrarily, a low level of self-efficacy in HCWs during the 2019-nCoV pandemic associates with heightened stress, anxiety, depressive symptoms, and insomnia (24) and is a risk factor for loneliness (51). A study on emergency room (ER) nurses found that goal orientation explains considerable variance in burnout and work engagement, with mastery-approach goal orientation being particularly beneficial (52). Health professionals using problem-solving-related strategies and positive re-assessment do not report any health problems and have a better emotional state than those employing other coping strategies (53). Challenge orientation negatively predicts burnout and secondary traumatic stress (compassion fatigue) in palliative care nurses (54). Thus, promoting this antibody can help reduce psychological distress and burnout in HCWs. Those with high social monitoring, mobilizing, and creating capacities have openness for contact with people, are socially assertive, and possess communication abilities. HCWs display reluctance to participate in the psychological interventions developed for them (55). Promoting these antibodies will benefit in inhibiting their hesitance to seek help or discuss their problems with a counselor or mental health professional. Synchronicity, impulse, emotion, and irritability control regulate emotions and prevent any emotional dysregulation or dissonance. The COVID-19 pandemic has made HCWs prone to emotional disturbances, vicarious trauma, and irritability (56, 57). Enhancing these antibodies, incredibly emotion and irritability control, in HCWs will enable them to use their frustration and anger constructively.

Table 1 provides some practical recommendations that can assist the HCWs in enhancing their PI and the intervention developers in designing effective PI-boosting interventions for HCWs. The medical workers with higher mental health problems report poor self-perceived physical health as well. Contrarily, the access to psychological aid (materials/ resources) is inversely related to the proportion of mental health problems (58). With this in view, researchers indicate the need for regular screening and timely addressal of psychological health concerns among HCWs, preferably through psychotherapeutic means (14, 59). As PI can be modified by psychotherapeutic interventions (34), developing evidence-informed, tiered, and tailored PI-boosting interventions will help protect the “protectors” from being victimized by the pandemic.

## Author Contributions

YA, AJ, and TS conceptualized the theme and prepared the final manuscript draft. AJ wrote the first draft. TS and YA reviewed and commented on the initial draft. All authors contributed to the article and approved the submitted version.

## Conflict of Interest

The authors declare that the research was conducted in the absence of any commercial or financial relationships that could be construed as a potential conflict of interest.
